# Bridging the gap between measurements and modelling: a cardiovascular functional avatar

**DOI:** 10.1038/s41598-017-06339-0

**Published:** 2017-07-24

**Authors:** Belén Casas, Jonas Lantz, Federica Viola, Gunnar Cedersund, Ann F. Bolger, Carl-Johan Carlhäll, Matts Karlsson, Tino Ebbers

**Affiliations:** 1 Division of Cardiovascular Medicine, Department of Medical and Health Sciences, Linköping University, Linköping, Sweden; 2 Center for Medical Image Science and Visualization (CMIV), Linköping University, Linköping, Sweden; 3 Department of Clinical Physiology, Department of Medical and Health Sciences, Linköping University, Linköping, Sweden; 4 Department of Biomedical Engineering, Linköping University, Linköping, Sweden; 5 Department of Medicine, University of California San Francisco, San Francisco, California USA; 6 Division of Applied Thermodynamics and Fluid Mechanics, Department of Management and Engineering, Linköping University, Linköping, Sweden

**Keywords:** Cardiology, Biomedical engineering, Mathematics and computing

## Abstract

Lumped parameter models of the cardiovascular system have the potential to assist researchers and clinicians to better understand cardiovascular function. The value of such models increases when they are subject specific. However, most approaches to personalize lumped parameter models have thus far required invasive measurements or fall short of being subject specific due to a lack of the necessary clinical data. Here, we propose an approach to personalize parameters in a model of the heart and the systemic circulation using exclusively non-invasive measurements. The personalized model is created using flow data from four-dimensional magnetic resonance imaging and cuff pressure measurements in the brachial artery. We term this personalized model the cardiovascular avatar. In our proof-of-concept study, we evaluated the capability of the avatar to reproduce pressures and flows in a group of eight healthy subjects. Both quantitatively and qualitatively, the model-based results agreed well with the pressure and flow measurements obtained *in vivo* for each subject. This non-invasive and personalized approach can synthesize medical data into clinically relevant indicators of cardiovascular function, and estimate hemodynamic variables that cannot be assessed directly from clinical measurements.

## Introduction

Currently, cardiovascular diseases are mainly assessed based on clinical markers at specific locations in the cardiovascular system. However, as a result of hemodynamic coupling, diseases are normally not limited to a single location but instead affect several sites in the heart and the vasculature. For example, in patients with aortic stenosis, the chronically elevated left ventricular afterload causes adverse cardiac remodelling over time. The detailed examination of single clinical biomarkers obscures the system-wide analysis demanded by the complex relationships in the cardiovascular system. Therefore, despite advances in imaging techniques and diagnostic parameters for characterizing hemodynamics, determining the overall multifaceted cardiovascular status of a specific patient remains a challenging task.

Alternatively, cardiovascular function can be studied using computational models. Models can give insight into the interactions between different parts of the cardiovascular system and provide a holistic approach, allowing us to better understand disease extent in individual patients. Furthermore, modelling approaches enable computation of hemodynamic variables that are difficult or impossible to measure experimentally, and allow for prediction of intervention outcomes. Among the different types of models, lumped parameter representations have been used extensively due to their ability to capture global hemodynamics while keeping computational demands low^1^. Relatively simple, fast models allow for real-time simulation and rapid feedback after changes in model parameters, which make them suitable for incorporation into clinical routine. Lumped parameter models of the cardiovascular system typically comprise multiple compartments that represent different parts of the heart and the vessels. Each compartment is defined by a set of parameters describing its mechanical properties, such as resistance to blood flow (R), blood flow inertia (L), and compliance (C). The complexity of the model depends on the specific research question and can vary between single-compartment representations, such as the well-known Windkessel model^2^ and more complex ones, such as the whole-body circulatory control system developed by Guyton *et al*.^3^.

The clinical applicability of computational models, including outcome predictions, is improved when such models are subject specific. This implies that the model should be established on the basis of physical principles that are assumed to be common to all subjects, but be capable of reproducing cardiovascular features observed experimentally by individual tuning of the system for each subject^4, 5^. Although lumped parameter models have long been used, starting with the mathematical formulation of the Windkessel model in 1899^2^, most authors have largely relied on generic population values of the parameters and have not attempted to render truly subject specific models. Only a few studies have combined clinical measurements with lumped parameter models to tailor them to a particular subject, but such approaches have so far required invasive measurements^6–10^ or can only identify a small number of parameters^11, 12^. Sughimoto *et al*.^8^ developed a closed-loop model of the cardiovascular system for application in the intensive care unit and tuned the five most sensitive parameters based on echocardiography data in the left ventricle and invasive catheter measurements of pressures and cardiac output. Keshavarz *et al*.^11^ introduced a simple model to compute left ventricular stroke work in patients with aortic stenosis which included the left ventricle and the systemic circulation. A subset of model parameters was estimated using echocardiography and 2D cine phase-contrast MRI (2D cine PC-MRI) flow data in the left ventricle and the heart valves. In the single-ventricle physiology model described by Pant *et al*.^10^, parameters were tuned with Doppler ultrasound and two-dimensional magnetic resonance flow measurements, as well as invasive pressure catheter measurements. Other authors have tuned parameters of three-element Windkessel models to achieve desired features of pressure and flow waveforms, and used these models as boundary conditions for three-dimensional blood flow simulations^4, 13^.

A current goal of cardiovascular modelling is the development of subject specific models that do not require invasive measurements, which would make them suitable for application in clinical routine. Three-dimensional, three-directional, cine phase contrast (PC) MRI, commonly referred to as 4D Flow MRI, is a promising technique to achieve this goal, as it enables access to a wide variety and quantity of data that has previously been unavailable or difficult to obtain. Unlike previous imaging modalities used for personalizing lumped parameter models, such as Doppler echocardiography and 2D cine PC-MRI, 4D Flow MRI permits retrospective flow assessment in all spatial directions and at any location within the imaging volume using data from a single acquisition.

Here, we present a proof-of-concept combination of 4D Flow MRI measurements and lumped parameter modelling to personalize a model of the cardiovascular system, which we term a cardiovascular avatar, based solely on non-invasive measurements.

## Results

### The cardiovascular avatar

**Figure 1:**
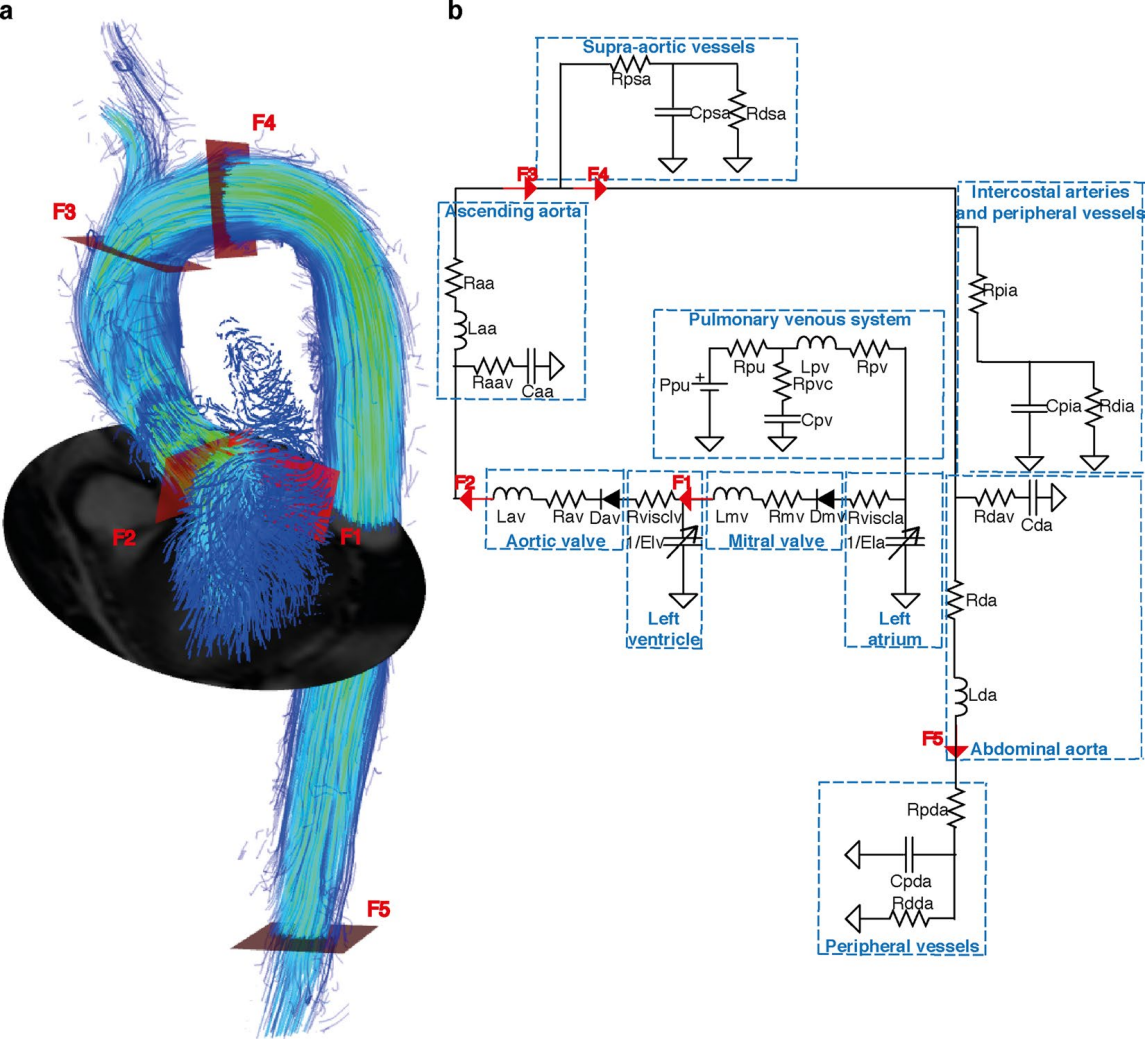
Illustration of the method to obtain a subject specific cardiovascular model. (**a**) Visualization of the 4D Flow MRI data in the heart and the aorta. Streamlines of the velocity field at peak systole are overlaid onto a segmentation of the left heart and the aorta. The positions of the analysis planes to extract the volumetric flow waveforms required by the model (F1-F5) are indicated as red planes. These positions correspond to: the mitral valve (F1); the aortic valve (F2); the ascending aorta, upstream from the brachiocephalic trunk (F3); the aortic arch (F4) and the abdominal aorta (F5). (**b**) A schematic diagram of the lumped parameter model, including the location of the flow measurements derived from the model indicated as red arrows. A description of the parameters in the model is given in Table 2. The parameters are adjusted such that the model can reproduce the flow waveforms obtained with 4D Flow MRI, as well as a number of cardiovascular indices obtained non-invasively.

The starting point for our model was the well-established lumped parameter model introduced by Sun *et al*.^14^. The model was subsequently modified according to measurements from common 4D Flow MRI analysis, which determine the level of detail that can be achieved in every compartment. To personalize the model, parameters were estimated using 4D Flow MRI and morphological MRI images as well as non-invasive pressure measurements in the brachial artery. The estimation of parameters was performed in two complementary steps. A small subset of parameters was initially computed based on a-priori information from the images and known values of cardiovascular indices. In a second step, the majority of the model parameters were estimated by minimizing the difference between flow profiles extracted from the 4D Flow MRI data at five predefined locations and those generated by the model. An overview of the approach is illustrated in Fig. 1.

### Comparison between model-based flow waveforms and *in vivo* measurements

**Figure 2:**
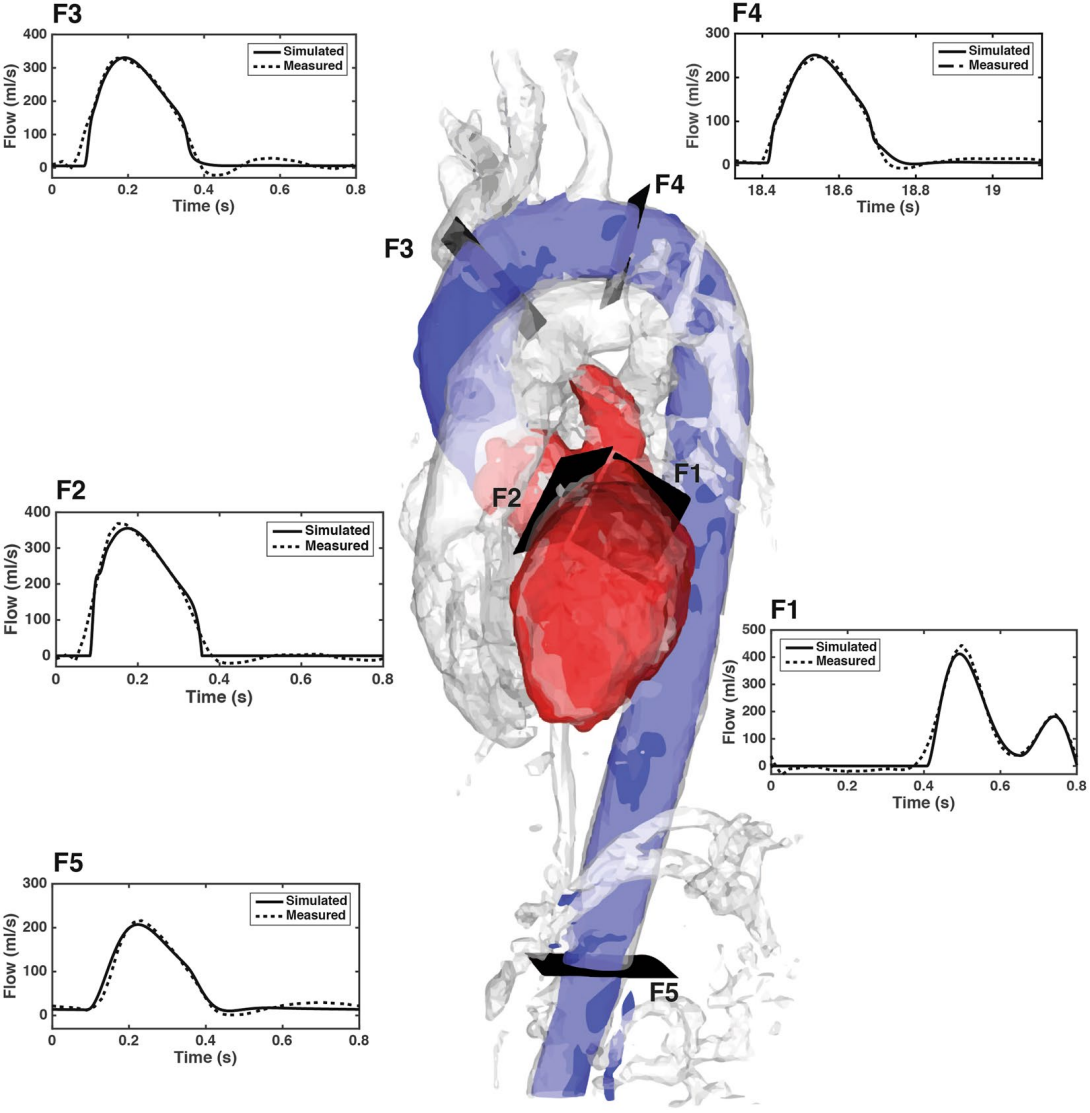
Comparison between model-based and measured volumetric flow waveforms for subject 1 after parameter estimation. Solid lines represent the flow waveforms generated by the model while dotted lines depict the flow waveforms measured with 4D Flow MRI. The flows correspond to five locations: the mitral valve (location F1), the aortic valve (location F2), the ascending aorta, upstream from the brachiocephalic trunk (location F3), the aortic arch (location F4) and the abdominal aorta (location F5). The subject specific geometry is represented by an angiography created from the 4D Flow MRI data. The anatomical regions of interest are highlighted using segmentations of the left heart (red) and the aorta (blue).

The proposed approach was applied to a group of eight healthy volunteers representing a spectrum of heart rates and arterial blood pressures (Table 1). Automatic parameter tuning to obtain a subject specific model was performed successfully for all subjects. A comparison between the model-based flow waveforms and the 4D Flow MRI measurements at the locations F1 to F5 for one of the volunteers (subject 1) after parameter estimation is shown in Fig. 2. The model-based flow waveforms showed good qualitative agreement with the measurements in terms of both wave shape and specific wave features. The model accurately characterized the mitral flow pattern (F1), including the amplitude and temporal location of the early and late filling phases (E wave and A wave, respectively) and the duration of diastasis. The amplitude and temporal location of the systolic peak in the flow at the aortic valve (F2) and the locations along the aorta (F3-F5) were also well reproduced by the model. Across all subjects, the mean relative differences between net flow measurements and model-based ones were 8%, 10%, 10%, 6% and 5% at locations F1 to F5, respectively.

**Table 1:** Characteristics of the subjects in the study.

	Study individuals (n = 8)
Gender	2 M, 6 F
Age, years	26 ± 2 (20–32)
Heart rate, bpm	67 ± 3 (55–82)
Systolic blood pressure (SBP), mmHg	113 ± 3 (98–125)
Diastolic blood pressure (DBP), mmHg	63 ± 3 (56–85)
Left ventricular end-diastolic volume (EDV), mL	152 ± 10 (119–199)
Left ventricular end-systolic volume (ESV), mL	69 ± 6 (42–98)
Stroke volume (SV), mL	83 ± 4 (70–103)

Values are expressed are mean ± standard error, unless otherwise stated. Ranges are given in parenthesis.

### Subject specific model output and comparison with measured variables

**Figure 3:**
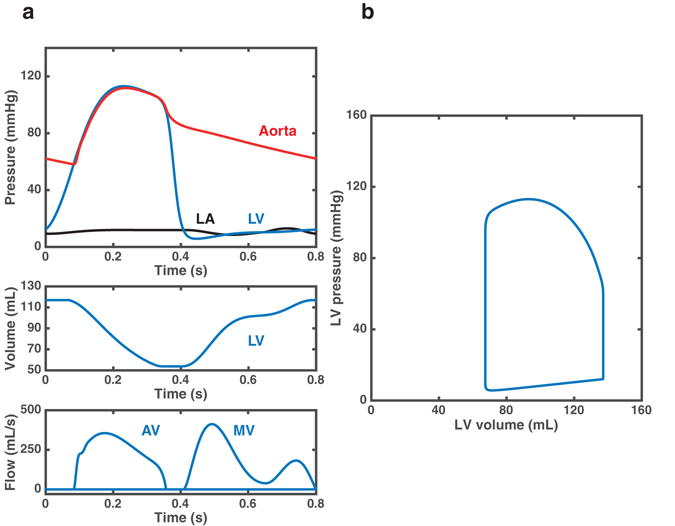
Model output including: (**a**) aortic root pressure (red), left ventricular pressure (blue), left atrial pressure (black) (top panel), left ventricular (LV) volume (middle panel) and flows through the mitral valve (MV) and the aortic valve (AV) (bottom panel); (**b**) left ventricular pressure-volume loop.

Clinically relevant output variables generated by the model after parameter estimation for subject 1 are shown in Fig. 3. The model reproduces realistic waveforms for several hemodynamic variables, including pressures in the left atrium, the left ventricle and the aorta, as well as blood volumes in the left ventricle and flows through the mitral and the aortic valve (Fig. 3a). Knowledge of left ventricular pressures and volumes allows for computation of the left ventricular pressure-volume loop, as shown in Fig. 3b. The simulated systolic and diastolic aortic pressure values were in good agreement with the non-invasive cuff measurements (112/58 vs. 114/56). The simulated and measured arterial pressures for all the subjects in the study are compared in Table 2. The SBP values predicted by the model for the subjects in the study (111.3 ± 5.1 mmHg) were in close agreement with the measurements (112.9 ± 3.8 mmHg), while predictions of DBP were, on average, slightly underestimated (54.3 ± 3.1 mmHg vs. 62.7 ± 3.4 mmHg).

**Table 2:** Comparison of measured and model-based SBP and DBP values for the eight subjects in the study. Measurements of SBP and DBP were obtained non-invasively in the brachial artery.

Subject number	SBP/DBP (mmHg)	
	Measured	Model-based
1	114/56	112/58
2	123/57	129/50
3	105/55	99/54
4	125/85	125/73
5	110/60	108/54
6	103/59	99/51
7	125/62	125/49
8	98/61	91/47

The model-based SBP and DBP correspond to the maximum and minimum of the model-based aortic pressure waveform, respectively.

### Results of subject specific parameter estimations

Table 3 shows a comparison between the model parameters obtained for all subjects after the optimization procedure and parameter values characterizing cardiovascular function in healthy adult subjects from previous studies^14–19^. The values reported by Sun *et al*.^14^ were calculated based on published data^20^ and Doppler ultrasound recordings of velocity patterns in the mitral valve and the pulmonary vein. The parameters describing the time-varying elastance function of the heart chambers in the work by Mynard *et al*.^15^ were calculated according to previous experimental studies on healthy subjects^21–23^. The arterial resistances reported by Liang *et al*.^17^ were chosen to obtain reasonable fits of model-based flows and 2D PC-MRI flow measurements along the aorta. The parameters estimated in our study are within the physiological range and consistent with these published values.

**Table 3:** Parameter estimates for the eight subjects included in the study. Values are given as mean ± standard error, unless otherwise stated.

Parameter	Description (units)	Literature values	Estimated values (n = 8)
**Pulmonary venous system**			
*P*_*pu*_	Pulmonary capillary pressure (mmHg)	7.4 (Sun *et al*.^14^)	10.8 ± 0.68
*R*_*pv*_^a^	Resistance of pulmonary veins (mmHg·s/mL)	2·10^−3^ (Sun *et al*.^14^)	2·10^−3^
*L*_*pv*_^a^	Inertance of pulmonary veins (mmHg· s^2^/mL)	5·10^−4^ (Sun *et al*.^14^)	5·10^−4^
*R*_*pvc*_^a^	Viscoelastic resistance of pulmonary capillaries and veins (mmHg·s/mL)	0.01 (Sun *et al*.^14^)	0.01
*C*_*pvc*_^a^	Capacitance of pulmonary capillaries and veins (mL/mmHg)	4 (Sun *et al*.^14^)	4
*R*_*pu*_^a^	Resistance of pulmonary capillaries (mmHg·s/mL)	0.01 (Sun *et al*.^14^)	0.01
**Heart parameters**			
**Left atrium (LA)**			
*K*_*s,LA*_	Source resistance coefficient of the LA (s/mL)	10·10^−9^ (Mynard *et al*.^15^)	12.26·10^−9^ ± 1.92·10^−9^
*E*_*min,LA*_	Minimal elastance of the LA (mmHg/mL)	0.08 (Mynard *et al*.^15^)	0.11 ± 3.5·10^−3^
*E*_*max,LA*_	Maximal elastance of the LA (mmHg/mL)	0.17 (Mynard *et al*.^15^)	0.15 ± 7.1·10^−3^
*V*_0,*LA*_	Unstressed volume of the LA (mL)	3 (Mynard *et al*.^15^)	2.68 ± 0.43
*m*_1_,_*LA*_	Contraction rate constant of the LA (−)	1.32 (Mynard *et al*.^15^)	1.22 ± 0.14
*m*_2_,_*LA*_	Relaxation rate constant of the LA (−)	13.1 (Mynard *et al*.^15^)	12.9 ± 1.96
*α*_1_,_*LA*_	Systolic time constant of the LA (−)	0.11 (Mynard *et al*.^15^)	0.1 ± 0.04
*α*_2_,_*LA*_	Diastolic time constant of the LA (−)	0.18 (Mynard *et al*.^15^)	0.2 ± 0.02
*R*_*visc*_,_*LA*_	Viscous loss resistance for the LA (mmHg·s/mL)	1·10^−4^ (Mynard *et al*.^15^)	1.37·10^−4^ ± 1.77·10^−5^
*Onset*_*LA*_	Onset of contraction of the LA (s)	0.85 (Mynard *et al*.^15^)	0.8 ± 0.01
**Left ventricle (LV)**			
*K*_*s*,*LV*_	Source resistance coefficient of the LV (s/mL)	4·10^−9^ (Mynard *et al*.^15^)	2.93·10^−9^ ± 4.35·10^−10^
*E*_*min*,*LV*_	Minimal elastance of the LV (mmHg/mL)	0.08 (Mynard *et al*.^15^)	0.09 ± 7.1·10^−3^
*E*_*max*,*LV*_	Maximal elastance of the LV (mmHg/mL)	3 (Mynard *et al*.^15^)	1.97 ± 0.17
*V*_0,*LV*_	Unstressed volume of the LV (mL)	10 (Mynard *et al*.^15^)	11.46 ± 2.03
*m*_1_,_*LV*_	Contraction rate constant of the LV (−)	1.32 (Mynard *et al*.^15^)	1.67 ± 0.12
*m*_2_,_*LV*_	Relaxation rate constant of the LV (−)	27.4 (Mynard *et al*.^15^)	33.92 ± 1.2
*α*_1_,_*LV*_	Systolic time constant of the LV (−)	0.269 (Mynard *et al*.^15^)	0.4 ± 0.03
*α*_2_,_*LV*_	Diastolic time constant of the LV (−)	0.452 (Mynard *et al*.^15^)	0.42 ± 0.01
*R*_*visc,LV*_	Viscous loss resistance for the LV (mmHg·s/mL)	1·10^−4^ (Mynard *et al*.^15^)	1.69·10^−4^ ± 1.62·10^−5^
*Onset*_*LV*_	Onset of contraction of the LV (s)	0 (Mynard *et al*.^15^)	−0.04 ± 8.8·10^−3^
**Mitral valve**			
*R*_*mv*_	Resistance of the mitral valve (mmHg·s/mL)	3.75·10^−3^ (Sun *et al*.^14^)	4.52·10^−3^ ± 7.46·10^−4^
*L*_*mv*_	Inertance of the mitral valve (mmHg·s^2^/mL)	2·10^−4^ (Sun *et al*.^14^)	7·10^−4^ ± 4.6·10^−5^
**Aortic valve**			
*EOA*_*av*_	Effective orifice area of the aortic valve (cm^2^)	1.69 (Garcia *et al*.^16^)	2.65 ± 0.54
*A*_*ao*_	Cross sectional area of the aorta (cm^2^)	5 (Olufsen *et al*.^62^)	6.09 ± 1.27
*L*_*av*_	Inertance of the aortic valve (mmHg· s^2^/mL)	4·10^−4^ (Sun *et al*.^14^)	2.74·10^−4^ ± 4.6·10^−4^
**Systemic arterial system**			
*R*_*aa*_	Resistance of the ascending aorta (mmHg·s/mL)	0.04 (Sun *et al*.^14^), 0.02 (Liang *et al*.^17^),	0.06 ± 0.03
*L*_*aa*_	Inertance of the ascending aorta (mmHg· s^2^/mL)	5·10^−4^ (Sun *et al*.^14^), 1.2·10^−3^ (Broome *et al*.^18^)	1.01·10^−4^ ± 1.06·10^−6^
*R*_*aav*_	Viscoelastic resistance for *C*_*aa*_ (mmHg·s/mL)	0.01 (Sun *et al*.^14^)	6.11·10^−3^ ± 1.8·10^−3^
*C*_*aa*_	Capacitance of the ascending aorta (mL/mmHg)	0.1 (Sun *et al*.^14^), 0.16 (Broome *et al*.^18^)	0.13 ± 0.01
*R*_*psa*_	Proximal peripheral resistance for the supra aortic vessels (mmHg·s/mL)	—	0.05 ± 3.5·10^−3^
*R*_*dsa*_	Distal peripheral resistance for the supra aortic vessels (mmHg·s/mL)	3.9 (Heldt *et al*.^19^)	3.47 ± 0.56
*C*_*psa*_	Peripheral compliance for the supra aortic vessels (mL/mmHg)	0.6 (Liang *et al*.^17^)	0.49 ± 0.03
*R*_*pia*_	Proximal peripheral resistance for the intercostal arteries (mmHg·s/mL)	—	0.05 ± 3.5·10^−3^
*C*_*pia*_	Peripheral compliance for the intercostal arteries (mL/mmHg)	0.93 (Liang *et al*.^17^)	0.13 ± 0.03
*R*_*dia*_	Distal peripheral resistance for the intercostal arteries (mmHg·s/mL)	3 (Heldt *et al*.^19^)	8.37 ± 1.18
*R*_*da*_	Resistance of the abdominal aorta (mmHg·s/mL)	0.04 (Sun *et al*.^14^) 0.02 (Liang *et al*.^17^)	0.07 ± 0.01
*L*_*da*_	Inertance of the abdominal aorta (mmHg· s^2^/mL)	5·10^−4^ (Sun *et al*.^14^), 1.6·10^−3^ (Broome *et al*.^18^)	1.7·10^−3^ ± 3.46·10^−4^
*R*_*dav*_	Viscoelastic resistance for *C*_*da*_ (mmHg·s/mL)	0.01	5.83·10^−3^ ± 6.36·10^−4^
*C*_*da*_	Capacitance of the abdominal aorta (mL/mmHg)	0.1 (Sun *et al*.^14^), 0.28 (Broome *et al*.^18^)	0.24 ± 0.04
*R*_*pda*_	Proximal peripheral resistance (mmHg·s/mL)	—	0.05 ± 6.4·10^−3^
*R*_*dda*_	Distal peripheral resistance (mmHg·s/mL)	1.2 (Sun *et al*.^14^), 1.31 (Broome *et al*.^18^)	0.91 ± 0.05
*C*_*da*_	Peripheral compliance (mL/mmHg)	2(Sun *et al*.^14^)	0.96 ± 0.11
**Other parameters**			
*T*^b^	Duration of the cardiac cycle (s)	—	0.9 ± 0.05
*ρ*	Density of blood (g/mL)	1.06	1.06

^a^Parameter values were assigned according to literature^14^.

### Parameter identifiability

The identifiability of the parameters for one of the study subjects (subject 1) was analyzed using the Profile Likelihood (PL) method^24^. Using this method, the confidence boundaries are characterized in each individual parameter direction. The approach is based on gradually varying the parameter under study while re-optimizing all the other parameters. The confidence boundary is found when the optimization fails to provide an acceptable agreement with the data. The resulting confidence intervals of the optimal parameters for subject 1 are summarized in Table 4.

**Table 4:** Estimated parameter values and the associated 95% confidence intervals [*σ*^−^, *σ*^+^] derived from the profile likelihood for subject 1.

Parameter	Estimated value	PL-based confidence interval		Identifiability
		*σ*^−^	*σ*^+^	
**Pulmonary venous system**				
*P*_*pu*_	12	11.07	12.92	Identifiable
**Heart parameters**				
**Left atrium (LA)**				
*K*_*s,LA*_	1.19·10^−8^	−∞	∞	Structurally non-identifiable
*E*_*min,LA*_	0.12	0.09	0.14	Identifiable
*E*_*max,LA*_	0.15	0.15	0.2	Identifiable
*V*_0,*LA*_	3.22	—	—	Practically non-identifiable
*m*_1,*LA*_	1.29	—	—	Practically non-identifiable
*m*_2,*LA*_	8.30	—	—	Practically non-identifiable
*α*_1_,_*LA*_	0.11	—	—	Practically non-identifiable
*α*_2_,_*LA*_	0.15		—	Practically non-identifiable
*R*_*visc*,*LA*_	8.46·10^−5^	−*∞*	*∞*	Structurally non-identifiable
*Onset*_*LA*_	0.75	0.75	0.85	Identifiable
**Left ventricle (LV)**				
*K*_*s,LV*_	2.09·10^−9^	−*∞*	*∞*	Structurally non-identifiable
*E*_*min,LV*_	0.10	0.06	0.14	Identifiable
*m*_1_,_*LV*_	1.66	1.48	1.82	Identifiable
*m*_2_,_*LV*_	35.89	27.54	49.29	Identifiable
*α*_1_,_*LV*_	0.41	0.30	0.50	Identifiable
*α*_2_,_*LV*_	0.48	0.38	0.58	Identifiable
*R*_*visc,LV*_		−*∞*	*∞*	Structurally non-identifiable
*Onset*_*LV*_	−0.06	−0.07	−0.06	Identifiable
**Mitral valve**				
*R*_*mv*_	6.26·10^−3^	—	—	Practically non-identifiable
*L*_*mv*_	5.9·10^−4^	2.67·10^−4^	—	Practically non-identifiable
**Aortic valves**				
*L*_*av*_	1.64·10^−4^	—	1.24·10^−3^	Practically non-identifiable
**Systemic arterial system**				
*R*_*aa*_	0.08	0.06	0.13^a^	Practically non-identifiable
*L*_*aa*_	1·10^−4^	—	1.46·10^−3^	Practically non- identifiable
*R*_*aav*_	5·10^−3^	−*∞*	*∞*	Structurally non-identifiable
*C*_*aa*_	0.11	0.02	0.21	Identifiable
*C*_*psa*_	0.31	0.14	0.31	Identifiable
*C*_*pia*_	0.06	−	0.29	Practically non-identifiable
*R*_*da*_	0.08	0.05	0.13^a^	Practically non-identifiable
*L*_*da*_	2.15·10^−3^	1.17·10^−3^	—	Practically non-identifiable
*R*_*dav*_	5·10^−3^	−*∞*	*∞*	Structurally non-identifiable
*C*_*da*_	0.21	0.07	0.34	Identifiable

^a^The confidence boundary is computed outside the parameter boundary used in the optimization. Empty confidence boundaries in one (or both) directions indicate that no crosses with the threshold were found within the parameter boundaries used for the optimization. Parameters with a confidence interval [**−***∞,∞*] had a flat profile likelihood for any arbitrary value of the parameter.

## Discussion

This work presents a proof-of-concept combination of a lumped parameter model and non-invasive measurements for personalizing a model of the left heart and the systemic circulation.

This presents an advantage over previous approaches to personalizing lumped parameter models, as they normally require invasive measurements^6–10^ or can only provide a small subset of subject specific parameters^11, 12^. The study demonstrated, in a group of eight healthy volunteers, that the flow waveforms reproduced by the model agree well with flow waveforms measured *in vivo* with respect to wave shape and specific wave features, and in terms of net flow volumes. The approach was evaluated by comparison of the simulated flow waveforms and arterial systolic and diastolic pressure values with their measured counterparts in the studied subjects. In addition, an identifiability analysis revealed that a number of clinically useful model parameters were determined with small uncertainty using the available measurements.

Other authors have estimated parameters in lumped parameter models by solving the inverse problem based on 2D cine PC-MRI and Doppler ultrasound flow waveforms^10, 25^. Solving the inverse problem can be a challenging task, especially if the measurements are sparse and there are a large number of parameters to be estimated. In this context, a significant advantage of 4D Flow MRI over the prior techniques is the considerably larger quantity and variety of data that can be obtained with a single acquisition. In studies using Doppler ultrasound measurements, there are often fewer measured waveforms available to estimate parameters in models of complexity comparable to ours^26^. Other approaches combine measurements from Doppler ultrasound and 2D cine PC-MRI, as well as invasive catheter pressure measurements, to obtain the data required for estimating the parameters in the model^10^. Another distinct advantage of 4D Flow MRI is the ability to perform retrospective analysis at any region within the acquisition volume. In our study, we retrospectively placed analysis planes at locations of interest and performed retrospective valve tracking. This may provide more accurate flow waveforms to solve the inverse problem, especially at the heart valves^27^, thereby improving the estimation of the parameters in the model. Moreover, the use of a 4D PC-MR angiogram enabled anatomical orientation, facilitating positioning of the analysis planes. This is particularly relevant in subjects with complex geometries, for whom Doppler measurements and positioning of the 2D PC-MRI planes are difficult to perform.

In a modelling framework, retrospective quantification is appealing as it allows retrieval and incorporation of new data into the model in an iterative manner. This in turn allows for modifications of the model without requiring new measurements. The model presented here included only the pulmonary venous system, the left heart, and the systemic arterial system. Given the large regional coverage that is possible with a 4D Flow MRI acquisition, the approach could easily be extended by extracting measurements at additional locations in the cardiovascular system as required by the model. The model would also be easily extendable to patients with a spectrum of cardiac, valvular, and vascular pathologies, whose effects could be assessed individually or in combination with other pathologies.

The model predicts SBP values with high accuracy as compared to reference non-invasive cuff measurements, while DBP predictions were, on average, slightly underestimated. This underestimation might in part be due to inaccuracies in estimated systemic compliances, since wave propagation phenomena (e.g. wave reflections) are not effectively represented when using a lumped parameter model. Previous studies have suggested that in young subjects, the reflected pulse wave returns in diastole due to a lower pulse wave velocity (PWV), thus increasing the DBP^28^. These findings could explain the underestimation in model-based DBP values since our study population only included young subjects. In future studies, the systemic circulation could be modelled following a one-dimensional approach to capture wave propagation effects in the arterial tree.

The initial values of the distal peripheral resistances in the supra-aortic vessels and the intercostal arteries are calculated assuming, as a simplification, that the mean pressure losses in the ascending aorta can be disregarded (i.e. the MAP at the aortic root equals the MAP at the inlet of the supra-aortic branches). However, as these represent only initial values and the peripheral resistances change during the optimization, the optimized resistance of the ascending aorta reflects pressure losses for the specific subject. Across all subjects, the mean pressure gradient across this resistance was in average ΔP = 6.37 mmHg. This value is in agreement with mean pressure gradients across the ascending aorta of healthy subjects computed using 4D Flow MRI^29^. Improvements of the current approach to estimate peripheral resistances could incorporate 4D Flow MRI-derived measurements of viscous dissipation^29^ and turbulent kinetic energy (TKE)^30^ to estimate pressure losses in the aorta directly from the 4D Flow MRI data.

The precision of the parameter estimation was assessed by computing confidence intervals using the PL method^24^. This method is generally more appropriate and provides better estimates of the confidence intervals than other commonly used approaches based on the analysis of the Hessian matrix^31^. Furthermore, as opposed to these approaches, the PL method allows to detect practical non-identifiabilities. Based on the PL analysis for one of the subjects in the study, 15 parameters in the model were determined with a finite confidence interval given the amount and quality of the available measurements (see Table 4). The majority of the parameters describing the left ventricle and several parameters in the systemic arterial system (e.g. the compliances and resistances), as well as the maximum and minimum elastances of the left atrium, were determined with a reasonably small uncertainty. Among the remaining parameters, 6 were found to be structurally non-identifiable and 11 practically non-identifiable.

Structural non-identifiabilities are associated with an over-parametrization of the model^32, 33^. The parameters describing the viscous losses in the left atrium and the left ventricle, as well as their source resistance coefficients and the viscoelastic resistances of the aortic segments were found to be structurally non-identifiable. These parameters could be set to a fixed value and potentially increase the idenfiability of the remaining parameters in the model. For instance, the values of the viscous resistances in the left atrium and the ventricle, *R*_*visc,LA*_, *R*_*visc,LV*_ could be estimated in a subject-specific manner using 4D Flow MRI viscous dissipation measurements^29^.

The identifiability analysis revealed that the parameters that determine the shape of the left atrial time-varying elastance function were practically non-identifiable. This suggests that the amount and/or quality of the measurement data were insufficient to estimate their values. Including new measurement data or improving image quality can in principle resolve practical non-identifiabilities^24^. Additional 4D Flow MRI-derived measurements in the left atrium, such as left atrial volumes and relative pressure differences^34^, could be incorporated in future studies to improve identifiability of left atrial parameters.

As a proof-of-concept study, the evaluation of this novel approach for generating a subject specific model was done in a small group of subjects. To further evaluate the approach, a study on a large patient cohort will be advantageous, preferably with comparison to invasive measurements. We found that the quality of the imaging data will impact the accuracy of the simulated variables. For example, mismatches between net flow volumes at the required locations in the 4D Flow MRI data will lead to inaccuracies in the estimated parameters, as the model obeys the law of conservation of mass. The accuracy of the measured flow waveforms is largely determined by the spatial and temporal resolution, as well as the signal-to-noise ratio (SNR). Selecting a VENC that is too low can cause aliasing, thereby leading to an incorrect quantification of the peak flow and net flow volumes. On the other hand, if the VENC is set too high, the SNR can be low in regions that are also of interest for the study (e.g. the heart). Typical VENC values are in the range 150–200 cm/s for the thoracic aorta and 100–150 cm/s for intra-cardiac flows^35^. In this study, we set the VENC to 140 cm/s to provide a good SNR in both the aorta and the heart, and used a phase-unwrapping algorithm. The voxel size was set to 2.8 × 2.8 × 2.8 mm^3^. Previous studies recommend a voxel size of 3 × 3 × 3 mm^3^ for flow quantification in the heart and great vessels^36^. Besides voxel size and SNR, sufficient temporal resolution is critical for characterizing the flow features over the cardiac cycle. We used retrospective cardiac gating with an effective temporal resolution of 40 ms, which should be sufficient for accurately characterizing the temporal variation of the flow waveforms^36^.

This study makes a leap forward by integrating non-invasive measurements from 4D Flow MRI, which can provide a large amount of information compared to previous imaging techniques, with a relatively simple model of the cardiovascular system. In the current model, several parameters were non-identifiable, either due to the structure of the model (i.e. structural non-identifiability) or the measurement data used for the estimation (i.e. practical non-identifiability). Including more data of sufficient quality will further increase the number of identifiable parameters. Furthermore, it should be possible to reduce the number of parameters to estimate, thereby increasing their reliability given the set of available measurements. The profile likelihood used in this study could be used to identify candidate parameters for model reduction, potentially yielding a minimal model that is completely identifiable^33^. It should be noted, however, that full identifiability is not a requirement for the use of the approach, as values of identifiable parameters are valid estimates even in models including non-identifiable parameters. Moreover, a model can generate well-determined predictions despite non-identifiability of some of its parameters^37^. Future studies should focus on improving the approach and evaluating whether the model parameters can be identified with an acceptable confidence interval for specific clinical applications, as well as the predictive power of the parameters under varying conditions in health in disease.

The identifiability analysis was performed under the assumption that the measurement noise had a standard deviation of one. An increased noise variance would most probably affect the confidence intervals of the estimated parameters, and therefore this should be taken into consideration when interpreting the reported confidence intervals. In addition to the input measurements, the model structure, the parameter bounds used in the optimization, and the assigned fixed parameters will affect identifiability, as well as the subject-specific parameter values. These constraints represent a priori information, in our case the assumption of parameters within physiological ranges for healthy subjects. The parameter values obtained using the proposed approach should therefore be interpreted in this context. Incorporating additional measurements into the model could avoid the need for assigning fixed values to parameters. Inferring parameter values from partial non-invasive measurements is a nontrivial process, which requires both appropriate observations and a model that reflects the underlying dynamics of the cardiovascular system. This implies an iterative approach, combining modifications in data analysis and model development. More research is needed to improve this combination and enhance the reliability of the conclusions that can be drawn from the model.

The novel integrated imaging-modelling approach presented here has the potential to add to the understanding of cardiovascular function in health and disease by allowing researchers and clinicians to extract the most relevant information from large and complex medical datasets, as well as enabling the estimation of hemodynamic features that are impractical to measure with current clinical methods. For instance, the analysis of pressure-volume loops could be possible without involving invasive catheter pressure measurements in the heart, which have associated risks and are difficult to perform. Furthermore, the approach could be used to present and combine multimodal cardiovascular functional measurement data in a generic manner in clinical and research applications. When only limited data is available, the PL method will facilitate guidance on uncertainty of the parameters and suggest additional measurements to improve this uncertainty. When sufficient data is available, the proposed approach could be valuable for predicting the outcome of various medical and procedural interventions and facilitating the process of patient-specific treatment planning.

In conclusion, the proposed approach allows for estimating subject-specific parameters in a lumped-parameter model of the heart and the systemic circulation based exclusively on non-invasive measurements. After personalization, the model can generate flow waveforms and arterial blood pressures that match measurements obtained *in vivo*. We believe that, with further validation, this approach could assist in the diagnosis of cardiovascular diseases and add to the process of treatment planning.

## Methods

### Study subjects and *in vivo* measurements

The characteristics of the healthy volunteers included in the study are shown in Table 1. Inclusion criteria were as follows: no history of cardiovascular disease, no medication for cardiovascular disorders and normal physical examination including normal arterial blood pressure at rest. Exclusion criteria were absence of normal ventricular size, wall thickness or wall motion based on cardiac MRI data. The study was performed in accordance with the declaration of Helsinki and approved by the Regional Ethical Review Board in Linköping. All subjects provided written informed consent before participation.

All subjects underwent MRI examinations on a clinical 3T scanner (Philips Ingenia, Philips Healthcare, Best, the Netherlands) to acquire 4D Flow MRI data and 2D cine balanced steady-state free-precession (bSSFP) morphological data. The 4D Flow MRI data were used for flow assessment, while morphological images were used for anatomical orientation and segmentation of the left ventricle. Systolic and diastolic blood pressures were measured non-invasively in the brachial artery five to ten minutes before the MRI scan using an oscillometric blood pressure monitor.

### MRI examinations and data processing

4D Flow MRI data were acquired during free breathing, using a navigator gated gradient-echo pulse sequence with interleaved three-directional flow-encoding and retrospective vector cardiogram controlled cardiac gating^38, 39^. Imaging parameters included: velocity encoding (VENC) 140 cm/s, flip angle 5°, echo time 3.0 ms, repetition time 5.2 ms, parallel imaging (SENSE) speed up factors of 3 (AP direction) and 1.6 (RL direction), k-space segmentation factor 2 and elliptical k-space acquisition. The spatial resolution was 2.8 × 2.8 × 2.8 mm^3^ and the temporal resolution approximately 40 ms. For a heart rate of 60 bpm, scan time was about 7–8 and 10–15 min excluding and including navigator efficiency, respectively. Following acquisition, the data were retrospectively reconstructed into 40 time frames and corrected for concomitant gradient fields on the scanner. Phase wraps were corrected offline using a temporal phase unwrapping method^40^. A weighted second-order polynomial fit to static tissue was used to correct for background phase errors^41^.

Morphological two-, three- and four-chamber long axis (LAx) and a stack of short-axis (SAx) images were acquired during end-expiratory breath holds using the following settings: echo time 1.4 ms, repetition time 2.8 ms, flip angle 45°. The bSSFP images were reconstructed into 30 time frames with a slice thickness of 8 mm. SAx images were reconstructed with a pixel size of 0.9 × 0.9 mm^2^ and the LAx images 0.83 × 0.83 mm^2^.

### The cardiovascular model

The lumped parameter model consists of three main compartments: the pulmonary venous system, the left side of the heart (including the left atrium, the mitral valve, the left ventricle and the aortic valve) and the systemic arterial system. The model of the systemic arterial system includes the ascending aorta, the aortic arch, the supra- aortic vessels, the descending thoracic aorta, the intercostal arteries and the abdominal aorta with their corresponding peripheral vascular beds. In the model, the brachiocephalic trunk, the left carotid and subclavian arteries, together with their vascular beds, are represented as a single compartment. A schematic diagram of the entire model is presented in Fig. 1. The parameters included in the model are listed in Table 3.

### Cardiac Model

#### The heart chambers

The contractile state of each chamber was modelled based on the time-varying elastance concept introduced by Suga *et al*.^42^. The time-varying elastance describes the relation between chamber pressure, *P*(*t*), and chamber volume, *V*(*t*), during a cardiac cycle:

1 $$P(t)=E(t)(V(t)-{V}_{0})(1-{K}_{s}q)\,({\rm{mmHg}})$$

where *V*_0_ is the unstressed volume, defined as the intercept of the end-systolic pressure-volume relationship with the volume axis^42^. The term *K*_*s*_*q* is an extension to the original formulation of the time-varying elastance and accounts for the dependence of chamber pressure on flow^15, 43, 44^. Viscous losses in the chambers during ejection were modelled by a linear resistance, *R*_*visc*_^45^.

The time course of the elastance, E(t), was represented by a “double-Hill”^22^ function:

2 $$E(t)=\alpha {E}_{max}(\frac{{(\frac{t}{{\alpha }_{1}T})}^{{m}_{1}}}{1+{(\frac{t}{{\alpha }_{1}T})}^{{m}_{1}}})(\frac{1}{1+{(\frac{t}{{\alpha }_{2}T})}^{{m}_{2}}})+{E}_{min}({\rm{mmHg}}/{\rm{ml}})$$

where *T* is the duration of the cardiac cycle and *E*_*max*_ and *E*_*min*_ the maximal and minimal elastance, respectively. *α*_1_ and *n*_1_ are dimensionless factors that determine the shape of the elastance during contraction, while *α*_2_ and *n*_2_ characterize relaxation. The scaling factor *α* ensures that the maximum of *E*(*t*) is *E*_*max*_.

#### The heart valves

The pressure gradient across the mitral valve, Δ*Pmv*, was described as:

3 $$\Delta {P}_{mv}(t)={R}_{mv}{Q}_{mv}(t)+{L}_{mv}\frac{d{Q}_{mv}(t)}{dt}({\rm{mmHg}})$$

where *Q*_*mv*_(*t*) is the instantaneous flow rate across the mitral valve and *Rmv* and *Lmv* represent the mitral valve resistance and inertance, respectively. The term *R*_*mv*_*Q*_*mv*_(*t*) characterizes pressure losses due to flow separation, while the second term in the equation accounts for the acceleration and deceleration of mitral flow^14^. Viscous losses were considered negligible and were therefore not included in the model.

The modelling of the aortic valve was based on the analytical description of the mean transvalvular pressure gradient derived by Garcia *et al*.^46^. This description introduces an energy loss coefficient, *E*_*L*_*C*_*O*_, to account for the well-known pressure recovery phenomenon^47^:

4 $$\Delta {P}_{av}(t)=\frac{\rho }{2{E}_{L}C{o}^{2}}{Q}_{av}^{2}(t)+\frac{2\pi \rho }{\sqrt{{E}_{L}Co}}\frac{d{Q}_{av}(t)}{dt}({\rm{mmHg}})$$

where *Q*_*av*_(*t*) is the instantaneous flow rate across the aortic valve and *ρ* the viscosity of blood. The energy loss coefficient is defined as *E*_*L*_*C*_*O*_ = (*EOA*_*av*_)*A*_*ao*_/(*A*_*ao*_ − *EOA*_*av*_), where *EOA*_*av*_ represents the effective orifice area of the valve and *A*_*ao*_ is the cross-sectional area of the aorta measured at the time of peak systole. In this formulation, the term accounting for flow acceleration is also a function of the energy loss coefficient. However, in the model described here, this term was characterized by a generic inertance *Lav* instead. The term $\frac{\rho }{2{E}_{L}C{o}^{2}}{Q}_{av}(t)$ characterizes the energy losses across the valve, represented by *R*_*av*_ in Fig. 1.

Pressure recovery is known to have an impact on the pressure gradient across the aortic valve^47^. For the mitral valve, however, this effect was ignored since the ratio between the effective valve area and the size of the left ventricle, which determines the magnitude of pressure recovery, is very small^48^.

Whether valves were considered as fully open or closed was dependent on the sign of the pressure gradient across them. The transition from closed to open state, which is modelled by a diode, is triggered by a forward pressure gradient.

### The pulmonary venous system and the systemic circulation

The complexity of the models of the pulmonary venous system and the systemic circulation was chosen based on the intended level of detail and the number of measurements available to characterize each region. The model for each vessel segment consists of a *RLCR*_*v*_ combination, where the resistance *R* accounts for frictional losses and *L* represents mass flow inertia. The combination of the resistance *R*_*v*_ and the compliance *C* models the viscoelastic properties of the vessel wall^14, 20^.

The pulmonary venous system was modelled by a constant pressure source *Ppu* representing perfusion pressure in the pulmonary capillaries and a vessel segment to characterize the pulmonary capillaries and veins, as previously described in the work of Sun *et al*.^14^. The aorta was divided into two segments, one of them corresponding to the ascending aorta, and a second segment including the descending part of the thoracic aorta and the abdominal aorta. Each aortic outlet (supra-aortic vessels and intercostal arteries) was coupled to a three-element Windkessel model representing the vasculature to the peripheral vascular bed, as previously described in other cardiovascular models^49, 50^. The Windkessel representation includes proximal and distal resistances (*Rp* and *Rd*, respectively) characterizing the resistance of the compartment and a compliance *Cp* to account for its total compliance. The peripheral resistance and compliance of the abdominal aorta were represented as in the original model by Sun *et al*.^14^.

### Image processing tools

Segmentation of the left ventricle in the Sax images was performed using the freely available segmentation software Segment version 1.9 (Medviso, Lund, Sweden)^51^. Images were visualized using commercially available visualization software (EnSight, CEI Inc., NC, USA). This software was used to compute the 4D PC-MR angiogram and place the analysis planes retrospectively. Valve tracking and computation of volume flow through the analysis planes were performed using in-house software written in Matlab (The Mathworks Inc., Natick, Massachusetts, USA).

### Computational aspects

The model equations were implemented in Matlab Simscape 2015b (The Mathworks Inc., Natick, Massachusetts, USA) and solved using the fixed step solver *ode14x* with a step size of 10^−3^ s. The extrapolation order and the number of Newton iterations were set to 1 and 4, respectively. The simulation started at the onset of isovolumetric contraction, with both the mitral and aortic valves closed. The end time for the simulation was set to 20 seconds, to ensure convergence of the solution.

### Subject-specific parameter estimation based on *in vivo* measurements

Estimation of the parameters in the model requires 4D Flow MRI derived measurements characterizing the morphology and function of the left ventricle and the aortic valve as well as volumetric flow waveforms from five sites (F1 to F5 in Fig. 1). These sites correspond to the mitral valve (F1), the aortic valve (F2), the distal ascending aorta, upstream from the brachiocephalic trunk (F3), the aortic arch (F4) and the abdominal aorta (F5).

The cardiovascular model has a total of 50 parameters, including 6 to represent the pulmonary venous system, 25 to model the heart and the valves and 17 for the systemic circulation. The length of the cardiac cycle (*T*) and the density of blood (*ρ*), are also defined as input parameters. All parameters in the model are listed in Table 3.

The selection of the parameters to be optimized was based on the availability of measurement data for a given model compartment. As there were no available measurements to characterize the pulmonary veins, the parameters in this compartment (*R*_*pu*_, *L*_*pv*_, *R*_*pv*_, *C*_*pvc*_, *R*_*pvc*_) were defined on the basis of values given by Sun *et al*.^14^. These parameters are expected to have little influence on the flow waveforms in the heart valves and the systemic circulation, thus they do not limit the subject specificity of the model. In addition, *ρ* was set to a constant value of 1.06 g/mL for all subjects. As a consequence, 44 of the initial 50 parameters were adjusted to be patient specific.

The estimation of the parameters to personalize the model was done in two consecutive stages. Initially, a subset of the parameters was estimated either from imaging-derived measures or by agreement with different cardiovascular indices, such as mean aortic pressure. This subset corresponds to the following 12 parameters:

$${\theta }_{measured}=\{EO{A}_{av},\,{A}_{ao},{R}_{psa},\,{R}_{pia},,\,{R}_{pda},\,{C}_{pda},\,{R}_{dda},\,T,\,{E}_{max,LV},{V}_{0,LV},\,{R}_{dia},\,{R}_{dsa}\}$$

Of these parameters, {*EOA*_*av*_, *A*_*ao*_, *R*_*psa*_, *R*_*pia*_, *R*_*pda*_, *T*} are fixed based on the measurements and do not change their value during the optimization procedure. *C*_*pda*_ and *R*_*dda*_ are not involved in the optimization. However, their values change as they are defined as a function of other compliances and resistances in the model. The parameters {*E*_*max*,*LV*_, *V*_0,*LV*_, *R*_*dia*_, *R*_*dsa*_} are included in the optimization with a range of variation of ±10%. In a second stage, nonlinear optimization was used to optimize the values of the remaining 32 parameters to obtain the best fit between the measured and the model-based flow waveforms.

### Initial parameter estimates

The cardiac cycle length, *T*, was approximated by the mean duration of the cardiac cycle during the 4D Flow MRI acquisition. The maximal elastance of the left ventricle *E*_*max*,*LV*_, i.e. the elastance value at the end of systole, was estimated as:

5 $${E}_{max,LV}=\frac{{P}_{es,LV}}{ESV-{V}_{0,LV}}({\rm{mmHg}}/{\rm{mL}})$$

where *P*_*es,LV*_ is the end-systolic pressure in the left ventricle, *ESV* the left ventricular end-systolic volume and *V*_0,*LV*_ the unstressed volume. *ESV* was calculated by manual segmentation of the endocardium in the short-axis stack at the time of end systole. *P*_*es,LV*_ was estimated from the brachial arterial systolic pressure (SBP) using equation (6), assuming that *P*_*es,LV*_ is related to the systolic pressure in the left ventricle, *P*_*s,LV*_, by *P*_*es,LV*_ = 0.9*P*_*s,LV*_^52^.

6 $${P}_{es,LV}=0.9({\rm{SBP}}+\Delta {P}_{av,max})({\rm{mmHg}})$$

The term Δ*P*_*av*,*max*_ accounts for the pressure drop across the aortic valve at peak systole, which can be computed using equation (4) in combination with the peak value of *Qav*(*t*) obtained from the 4D Flow MRI measurements. *ESV* was set to the value derived from the 4D Flow MRI data and *V*_0,*LV*_ was defined based on a preliminary estimation of the end-systolic elastance obtained using the non-invasive method described by Chen *et al*.^53^. The value of the ejection fraction (EF) involved in the calculation was computed from the estimated value of *ESV* and the time integral of the flow through the aortic valve. The ratio of pre-ejection period to total systolic period was estimated based on the timings inferred from the flow waveform at the aortic valve, derived from the 4D Flow MRI data.

The systemic vascular resistance (SVR) was computed as the mean arterial pressure (*MAP*) over the cardiac output (CO)^11, 54^. *MAP* was estimated from the brachial systolic and diastolic pressures (SBP and DBP, respectively) as given below^55^:

7 $$MAP=DBP+[\frac{1}{3}+(HR\cdot 0.0012)](SBP-DBP)({\rm{mmHg}})$$

The distal Windkessel resistances in the supra-aortic vessels (*R*_*dsa*_) and the intercostal arteries *R*_*dia*_ were computed based on the MAP value and the time-averaged flow rate at each outlet, obtained from the 4D Flow data. The peripheral resistance corresponding to the abdominal aorta, *Rdda*, was defined as a function of the resistances included in the systemic arterial system to achieve the intended value of SVR^11^. The proximal Windkessel resistances were set to match the characteristic impedance of the feeding vessel segment, as previously described by Garcia-Canadilla *et al*.^25^. The peripheral compliances of the supra-aortic vessels (*C*_*dsa*_) and the intercostal arteries (*C*_*pia*_) were estimated based on the pulse pressure (PP) derived from the brachial pressure measurements and the measured time-averaged flow rates^11^. The peripheral compliance of the abdominal aorta (*C*_*pda*_) was adjusted to match the estimated total compliance of the system^56^. Initial values for the remaining parameters in the models were established according to standard physiological considerations or values reported in previous studies^14, 15, 57^.

### Nonlinear optimization

Nonlinear optimization was used to estimate parameter values by minimizing the sum squared error (SSE) between the flow waveforms obtained from the 4D Flow MRI data and those generated by the model. The criterion function *J*was defined as the sum of the individual errors from each waveform:

8 $$J(p)=\mathop{\sum }\limits_{i=1}^{5}\mathop{\sum }\limits_{t=1}^{N}{(\frac{{Q}_{i}(t)-\tilde{{Q}_{i}}(t,p)}{{\sigma }_{i}(t)})}^{2}$$

where *i* is summed over the five locations where the flow waveforms *Q*_*i*_(*t*) are measured, $\tilde{{Q}_{i}}(t)$ represent the waveforms generated by the model as predicted by the parameters *p*and N is the number of time points of each flow waveform. The standard deviation of the measurement noise, *σ*_*i*_(*t*), was assumed to be 1.

Model parameters were estimated using the Levenberg-Marquardt algorithm^58^, which is an iterative, nonlinear least-squares optimization method. The optimization routine was terminated when parameter values in consecutive iterations changed by less than 0.1%^59^. The algorithm provides a set of optimized parameter values that represent a local solution to the optimization problem. To speed up the simulations, a subset of parameter values was initially estimated with the available non-invasive data, as described in the previous section. Furthermore, intervals of parameter values were defined to ensure that the solution was within the physiological range. For an initial parameter value of p_0_ in a certain parameter, these intervals were defined as follows: [p_0_/2, 2p_0_] for resistance components and [p_0_/5, 5p_0_], [p_0_/6, 6p_0_] for inertance and compliance components, respectively. Parameters characterizing the left atrium and the left ventricle were restricted to the interval [p_0_/2, 2p_0_].

### Data analysis

Flows through the heart valves were calculated using retrospective valve tracking with correction for through-plane motion^27^. For extracting flows along the aorta, a 4D PC-MR angiogram was derived from the 4D Flow MRI data to identify the locations of the analysis planes F3 to F5. To compute volume flow through each plane, the vessel contour or the valve orifice were manually segmented for every cardiac time frame^60^. At time frame *tf*, the segmented area included a set of pixels. The flow through a pixel *i* at the given time frame was computed as:

9 $${Q}_{i}({t}_{f})=v{\perp }_{i}\cdot {a}_{i}({{\rm{m}}}^{3}{s}^{-1})$$

where *v*⊥_*i*_ is the component of the velocity vector perpendicular to the plane in pixel *i* and *a*_*i*_ the area of the pixel. The volumetric flow through the segmented area *Q*(*t*_*f*_) was then calculated as follows

10 $$Q({t}_{f})=\mathop{\sum }\limits_{i=1}^{N}{Q}_{i}({t}_{f})({{\rm{m}}}^{3}{s}^{-1})$$

where N is the number of pixels included in the segmentation.

Input model parameters describing left ventricular and aortic valve function were also derived from the 4D Flow MRI data. The end-systolic volume of the left ventricle (ESV) was computed by manual segmentation of the endocardium in the SAx images at the time of end systole. The cross-sectional area of the aorta was calculated by manually segmenting the aortic lumen contour in the 4D Flow MRI data at peak systole. The measurement was performed distal to the coronary artery ostia and the aortic cross-sectional area was assumed to be circular. The effective orifice area of the aortic valve, *EOA*_*av*_, was approximated based on the continuity equation, as previously described by Garcia *et al*.^61^:

11 $$EO{A}_{av}=SV/VT{I}_{av}$$

where SV is the stroke volume, calculated as the time integral of the flow through the aortic valve, and *VTI*_*av*_ is the velocity-time integral of the instantaneous velocity at the center of the valve.

### Identifiability analysis

The identifiability analysis was performed using the PL algorithm^24^, in order to assess structural and practical identifiability and calculate confidence intervals for the parameter estimates. The threshold for the algorithm was calculated for a confidence level of 95% and standard deviation of the measurement noise equal to 1. Based on the article by Raue *et al*.^24^, a parameter is practically identifiable if its confidence boundaries are non-infinite in both directions. For practically non-identifiable parameters, the confidence boundary extends infinitely in one of the directions, although the profile likelihood has a unique minimum. Structurally non-identifiably parameters, on the other hand, have an infinite confidence interval (i.e. the profile likelihood is flat). Parameters with a finite confidence interval, and at least one confidence boundary outside the parameter range used in the original optimization are also considered as practically non-identifiable in this work.

### Data availability

The MRI datasets used to construct the models are available from the Linköping University Hospital for researchers who meet the criteria for access to confidential data. The IRB form states that the data obtained from the patients will be stored on secure computers within the Linköping University Hospital. The codes used for simulating the cardiovascular model, as well as the optimization algorithms for personalization, are available from the author upon request.
