# Publisher Correction: Bridging the gap between measurements and modelling: a cardiovascular functional avatar

**DOI:** 10.1038/s41598-020-58809-7

**Published:** 2020-01-29

**Authors:** Belén Casas, Jonas Lantz, Federica Viola, Gunnar Cedersund, Ann F. Bolger, Carl-Johan Carlhäll, Matts Karlsson, Tino Ebbers

**Affiliations:** 1 Division of Cardiovascular Medicine, Department of Medical and Health Sciences, Linköping University, Linköping, Sweden; 2 Center for Medical Image Science and Visualization (CMIV), Linköping University, Linköping, Sweden; 3 Department of Clinical Physiology, Department of Medical and Health Sciences, Linköping University, Linköping, Sweden; 4 Department of Biomedical Engineering, Linköping University, Linköping, Sweden; 5 Department of Medicine, University of California San Francisco, San Francisco, California USA; 6 Division of Applied Thermodynamics and Fluid Mechanics, Department of Management and Engineering, Linköping University, Linköping, Sweden

Correction to: *Scientific Reports* 10.1038/s41598-017-06339-0, published online 24 July 2017

In the original version of the Article, the following paragraph was omitted from the beginning of the Introduction:

“Currently, cardiovascular diseases are mainly assessed based on clinical markers at specific locations in the cardiovascular system. However, as a result of hemodynamic coupling, diseases are normally not limited to a single location but instead affect several sites in the heart and the vasculature. For example, in patients with aortic stenosis, the chronically elevated left ventricular afterload causes adverse cardiac remodelling over time. The detailed examination of single clinical biomarkers obscures the system-wide analysis demanded by the complex relationships in the cardiovascular system. Therefore, despite advances in imaging techniques and diagnostic parameters for characterizing hemodynamics, determining the overall multifaceted cardiovascular status of a specific patient remains a challenging task.”

This Article also contained an error in the Methods section under subheading ‘MRI examinations and data processing’,

“Imaging parameters included: velocity encoding (VENC) 140 cm/s, flip angle 5°, echo time 3.0 ms, repetition time 5.2 ms, parallel imaging (SENSE) speed up factors of 3 (AP direction) and 1.6 (RL direction), k-space segmentation factor 3 and elliptical k-space acquisition.”

now reads:

“Imaging parameters included: velocity encoding (VENC) 140 cm/s, flip angle 5°, echo time 3.0 ms, repetition time 5.2 ms, parallel imaging (SENSE) speed up factors of 3 (AP direction) and 1.6 (RL direction), k-space segmentation factor 2 and elliptical k-space acquisition.”

This Article also contained an error in Equation 8,

$$J(p)={\sum }_{i=1}^{5}{\sum }_{t=1}^{N}{\left(\frac{{Q}_{i}\left(t\right)-\tilde{{Q}_{i}}\left(t,p\right)}{{\sigma }_{i}{(t)}^{2}}\right)}^{2}$$

now reads:

$$J(p)={\sum }_{i=1}^{5}{\sum }_{t=1}^{N}{\left(\frac{{Q}_{i}\left(t\right)-\tilde{{Q}_{i}}\left(t,p\right)}{{\sigma }_{i}(t)}\right)}^{2}$$

These errors have now been corrected in the PDF and HTML versions of the Article.

